# Total Lignans of *Schisandra chinensis* Ameliorates Aβ_1-42_-Induced Neurodegeneration with Cognitive Impairment in Mice and Primary Mouse Neuronal Cells

**DOI:** 10.1371/journal.pone.0152772

**Published:** 2016-04-01

**Authors:** Xu Zhao, Chunmei Liu, Mengjie Xu, Xiaolong Li, Kaishun Bi, Ying Jia

**Affiliations:** 1 School of Traditional Chinese Materia Medica, Shenyang Pharmaceutical University, Wenhua Road 103, Shenyang, 110016, China; 2 School of Pharmacy, Shenyang Pharmaceutical University, Wenhua Road 103, Shenyang, 110016, China; Sungkyunkwan University, REPUBLIC OF KOREA

## Abstract

Lignan compounds extracted from *Schisandra chinensis* (Turcz.) Baill. have been reported to possess various biological activities, and have potential in the treatment of Alzheimer’s disease. This study was designed to investigate the effects of total lignans of *Schisandra chinensis* (TLS) on cognitive function and neurodegeneration in the model of AD induced by Aβ_1–42_ *in vivo* and *in vitro*. It was found that intragastric infusion with TLS (50 and 200 mg/kg) to Aβ_1–42_-induced mice significantly increased the number of avoidances in the shuttle-box test and swimming time in the target quadrant in the Morris water maze test. TLS at dose of 200 mg/kg significantly restored the activities of total antioxidant capacity (T-AOC), as well as the level of malondialdehyde (MDA) both in the hippocampus and cerebral cortex in mice. Results of histopathological examination indicated that TLS noticeably ameliorated the neurodegeneration in the hippocampus in mice. On the other hand, TLS (100 μM) could protect the Aβ_1–42_-induced primary mouse neuronal cells by blocking the decrease of mitochondrial membrane potential (MMP), change the expressions of Bcl-2 (important regulator in the mitochondria apoptosis pathway). Moreover, TLS also decreased the activity of β-secretase 1 (BACE1), crucial protease contributes to the hydrolysis of amyloid precursor protein (APP), and inhibited the expression of JKN/p38, which involved in the MAPKs signaling pathways in both mice and primary mouse neuronal cells. In summary, TLS might protect against cognitive deficits and neurodegeneration by releasing the damage of oxidative stress, inhibiting the expression of BACE1 and the MAPKs inflammatory signaling pathways.

## Introduction

Alzheimer’s disease (AD) is the most frequent type of neurodegenerative disorder in the elderly brain [1, 2], which is characterized by progressive neuronal loss, intracellular neurofibrillary tangles, and extracellular β amyloid (Aβ) deposits [3]. AD interrupts various functions of the brain like memory, intelligence, judgment and learning abilities [4]. Stunningly, about one-third of older adults at the time of death meet pathologic criteria for possible or probable AD [5, 6]. Therefore, an agent could prevent or slow down the progression of AD is necessary.

For the multifactorial implications of AD type neurodegeneration, Aβ is one of the underlying mechanisms [7]. Both formation and accumulation of Aβ were involved in the neurodegeneration associated with AD [8–10]. The Aβ peptide is derived from sequential cleavage of the amyloid precursor protein (APP). Two proteases contribute to the hydrolysis of APP, referred to γ- and β-secretase (β-site amyloid precursor protein cleaving enzyme 1, BACE1). γ-secretase cleaves a short C-terminal fragment of APP [11], and β-secretase cleaves in the ectodomain of APP at the N-terminus of the Aβ domain [12], which both lead to Aβ secretion. Increasing APP proteolytic processing based on gene therapy, transcriptional activation or even pharmacological activation of APP-degrading enzymes has represented a novel therapeutic strategy for the treatment of AD, which is currently being evaluated in cell-culture and animal models [13–15].

Recently, natural products have been served as the source for a large fraction of drugs and active compounds [16–18]. *Schisandra chinensis* (Turcz.) Baill. has been used as a tonic for kidney yin deficiency in traditional Chinese medicine for a long history. During recent decades, there are many reports about active lignan components [19–23] of *Schisandra chinensis* ameliorating cognition in models with dementia. However, there are still few research focusing on mechanisms underlying the effects of total lignans of *Schisandra chinensis* (TLS) in both cells and tissues induced by Aβ_1–42_. In the present study, we investigated the effect of TLS on Aβ_1–42_-induced neurodegeneration with cognitive impairment both in the mouse and primary mouse neuronal cell of AD models. Biochemical analyses and histopathological examination were used to represent the potential mechanisms.

## Materials and Methods

### Ethics Statement

This study was conducted in accordance with the Guidelines for Animal Experimentation and the protocol was approved by the Animal Ethics Committee of Shenyang Pharmaceutical University. All the cells and tissues of the mice were authorized to scientific purpose.

### Chemicals, reagents and materials

*Schisandra chinensis* (Turcz.) Baill. was purchased from Decaotang Traditional Pharmacy (Shenyang, China). The crude drug was identified and evaluated by Professor Ying Jia, expert of traditional Chinese medicine appraisal in the school of traditional Chinese materia medica of Shenyang Pharmaceutical University. Aβ_1–42_ peptide was purchased from Sigma-Aldrich (St Louis, MO, USA), and dissolved in physiological saline to a stock concentration of 1.0 mg/ml. The solution of Aβ_1–42_ was incubated at 4°C for 24 h to obtain the oligomeric form (for primary neuron cells tests) or incubated at 37°C for 5 d to gain agglomerative form (for *in vivo* tests)_,_ respectively. Donepezil was provided by Eisai China lnc. (Shanghai, China). Dulbecco’s modified eagle’s medium (DMEM) and fetal bovine serum (FBS), B27 supplement and 0.25% trypsin were purchased from Hyclone (Logan, UT, USA). 3-(4, 5-dimethylthiazol-2-yl)-2, 5-diphenyltetrazolium bromide (MTT), dimethyl sulphoxide (DMSO), rhodamine 123, penicillin and streptomycin were obtained from Sigma (St. Louis, MO, USA). The assay kits of total antioxidant capacity (T-AOC), malondialdehyde (MDA), superoxide dismutase (SOD), β-secretase 1 (BACE1), Aβ_1–42,_ B-cell lymphoma-2 (Bcl-2), c-Jun N-terminal kinase (JNK) and p38 were purchased from Nanjing Jiancheng Bioengineering Institute (Nanjing, China).

Male KM mice, weighing 25–30 g were purchased from the Central Animal House of Shenyang Pharmaceutical University (Shenyang, China). Mice were housed in groups of 6 per cage, allowed access to water and food ad libitum, and maintained in constant temperature (23 ± 1°C) and humidity (55 ± 5%) under a 12 h light/dark cycle (lights on 07:00 to 19:00 h).

### Preparation of TLS

The air-dried fruits of *Schisandra chinensis* were crushed into powder. The sample was weighed and refluxed with 95% ethanol (1:10 *w/v*) three times (each time for two hours). The solvent was evaporated under reduced pressure, and the extract was dispersed with 30% ethanol. The suspension was then subjected to chromatographic separation on the prepared AB-8 macroporous adsorption resin (Tianjin, China). 30% EtOH-H_2_O was used to except the large polar substances, and then 70% EtOH-H_2_O was used to concentrated the total lignans. The fraction of 70% EtOH-H_2_O was concentrated under reduced pressure and dried in vacuum to give the purified product.

The content of TLS of the extract was determined by UV spectrophotometry. Schisandrin was used as a reference standard to represent the total lignans content. The absorbance (A) was measured at the maximum collaborative absorption of 252 nm with 100% methanol as blank control. Extraction rate of the content of total lignans from *Schisandra chinensis* was 7.93%, and the purity of total lignans by purification was 68.72%.

### UPLC–Q-TOF/MS analysis of TLS chemical composition

**Fig 1:**
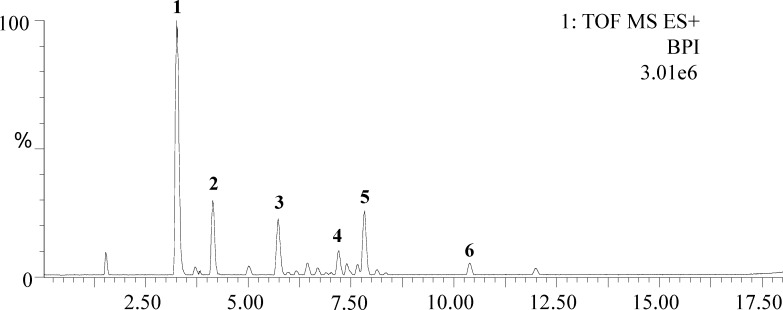
Chromatogram of TLS analyzed by UPLC-Q-TOF/MS with UV detection at 216 nm. The analysis was performed with an HSS T3 column (2.1 mm × 100 mm, 1.8 μm) at 30°C. Compounds were eluted (mobile phase A: water; B: acetonitrile) at a flow rate of 0.5 ml/min using a gradient program. Representative chromatographic peak: (1) Schisandrin, (2) Schisandrol B, (3) Angeloylgomisin H, (4) Schisantherin A, (5) Schisantherin B, (6) Deoxyschisandrin.

TLS was qualitatively analyzed employing UPLC-Q-TOF/MS method which analysis was carried out on an Acquity^TM^ ultra-performance liquid chromatography and a Waters Q-TOF (Waters Corp., Manchester, U.K.) equipped with an electrospray ionization (ESI) source. An Acquity Binary Manager system and an auto-sampler were used for the UPLC-Q-TOF/MS analysis. Data were performed with Masslynx V4.1 software. As shown in Table 1, twelve lignans were tentative identified by the full scan on the positive ion mode of MS/MS analysis. Six main compounds of those lignans were identified with the retention times and UV spectra of the reference substance, illustrated in Fig 1.

**Table 1:** Tentative identification of the compounds from TLS.

No.	Compound name	Retention time (min)	Molecular weight	Selected ion (m/z)
1	schisandrin	3.26	433.2226	[M+Na] ^+^ 455.2020
2	gomisin D	3.71	531.2230	[M+Na] ^+^ 554.2076
3	schisandrol B	4.14	417.1913	[M+H-H_2_O] ^+^ 399.1750
4	tigloylgomisin H	5.02	501.2248	[M+Na] ^+^ 524.2336
5	pregomisin	5.23	391.2121	fragment ion
6	angeloylgomisin H	5.73	501.2248	[M+Na] ^+^ 524.2322
7	Gomisin K_1_	7.03	403.2121	fragment ion
8	Gomisin K_3_	7.21	403.2121	fragment ion
9	schisantherin A	7.40	537.2125	[M+H] ^+^ 583.2126
10	schisantherin B	7.83	514.2281	fragment ion
11	gomisin E	8.86	514.2281	[M+Na] ^+^ 537.2086
12	deoxyschisandrin	10.38	417.2277	[M+Na] ^+^ 440.2079

The mass spectrometer was operated in positive ionization mode with the capillary voltage at 2.5 kV, and the cone voltage was set to 35 V. Source and desolvation temperatures were set at 130 and 450°C, respectively. The cone and desolvation gas flows were 50 and 800L/h, respectively.

### Treatments of the mice

**Fig 2:**
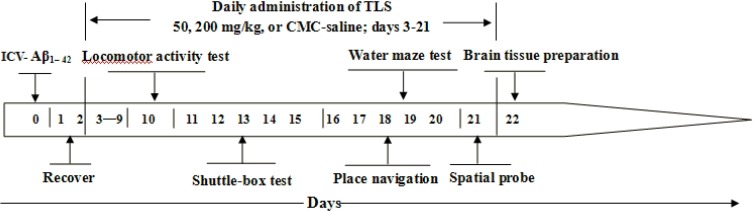
Experiment schedule.

The mice were randomly divided into five groups of 12 individuals each: (1) Control group (Aβ_1–42_-lesioned plus intragastric (i.g.) infusion carboxyl-methylcellulose (CMC) saline solution-treated), (2) Sham group (saline-lesioned plus i.g. infusion CMC-saline solution-treated), (3) TLS 50 mg/kg treated group (Aβ_1–42_-lesioned plus i.g. infusion TLS 50 mg/kg/day-treated), (4) TLS 200 mg/kg treated group (Aβ_1–42_-lesioned plus i.g. infusion TLS 200 mg/kg/day-treated), and (5) Donepezil treated group (Aβ_1–42_-lesioned plus i.g. infusion donepezil 3 mg/kg/day-treated). Mice in the control, donepezil, and TLS (50, 200 mg/kg) treated groups were anesthetized with chloralhydrate (200 mg/kg body weight, i.p.) and injected with aggregated Aβ_1–42_ peptide (3 μl) into the left lateral ventricle (AP, −0.5 mm, ML, −1.1 mm, DV, −3.0 mm) according to a mouse brain atlas [24]. Mice in the sham group were injected with physiological saline in the same area. Microinjections were performed by the 5-μl Hamilton syringe. After the lesion mice were received penicillin-G 200,000 IU/ml (0.2 ml/mouse, single dose, intramuscular). Then they were allowed to recover for 3 days before i.g. infusion of total lignans, donepezil or CMC-saline. Behavioral tests were started after 7 days of treatment. The health status (body weight and eating situation) of the mice were monitoring every day, and no animal died unexpectedly during any of the behavioral tests, or drug treatment. The experiment schedule is shown in Fig 2.

### Locomotor activity test

Spontaneous locomotor activity was measured using an animal activity cage with infrared sensitive photocells. Before locomotor test, animals were placed individually in the actophotometer cage for 2 min for habituation. Thereafter, locomotor activity was recorded for a period of 5 min. During this period, locomotor activity and immobility time were recorded.

### Active avoidance test

An automatic reflex conditioner (shuttle box) for active avoidance was used. All avoidance training sessions consisted of 30 trials with the following parameters: 10 s buzzer (670 Hz and 70 dB), 10 s foot shock (0.4 mA), and 10 s pause. Before the first trial of learning session, the mice were received 5 min to ambulate in the shuttle-box in order to be familiarized with the learning environment. A memory retention session with the same parameters without foot shock, after the 4 consecutive days of learning, was performed. The parameter automatically counted was the number of conditioned responses (avoidances).

### Morris water maze test

A spatial learning and memory test was performed by the method of Morris [25] with minor difference. Water maze test was consisted of a place navigation test and a probe test. The place navigation test was performed as two trials daily for 5 consecutive days. Mice were trained to locate and escape onto the platform during this session. A different starting position for each mouse was used in each trial. The mice were allowed to swim freely to find the hidden platform within 90 s. Animals failed to find the location within the given time were gently guided to the platform and were allowed to stay on it for 20 s and then returned to the cage. For each individual mouse, the position of the platform was fixed throughout the test. The escape latency, swimming distance and swimming velocity of each mouse per day were calculated. On the memory retention session (probe test), a single test was conducted in the pool which the platform was removed. The time spent in the target quadrant (southeast) and the times crossed where the platform was originally located were measured for each mouse.

### Brain tissue preparation

On the day after behavioral tests, all the mice were sacrificed by cervical dislocation and the brain was immediately removed. The hippocampus and cerebral cortex of 8 mice in each group were dissected out, and stored at −80°C until the biochemical studies. The entire brain of the other mice in each group were fixed in 10% formalin at 4°C and then embedded in paraffin for histopathological analysis.

### Biochemical analysis

Before detection, each part of the brain tissue was rapidly homogenized in ice-cold saline and the homogenates were centrifuged at 3500 rpm at 4°C for 15 min. The supernatant was collected for assay. The hippocampus and cerebral cortex tissue supernatant were used to measure the activities of antioxidative enzymes including SOD, BACE1, and T-AOC as well as the levels of MDA and Aβ_1–42_ by means of the assay kits according to the manufacturer's directions.

### Histopathological examination

For histopathology, the entire brains were postfixed in 4% paraformaldehyde (PFA) solution for 48 h, then transferred to 30% sucrose in 0.1mol/l PBS (pH 7.4) for at least 16 h until they sank for cryoprotection. The hippocampuses were then kept in the final sucrose solution until sectioning. Serial (neighboring) sections of 10 μm thickness were cut and stained with haematoxylin and eosin (H and E) [26].

### Culture of primary mouse neuron cells

Primary neuron cells were obtained from the cortex and hippocampus of newborn KM mice (0–1 day). After dissection and removal of the meninges, the cortices and hippocampus were minced and dissociated with 0.25% trypsin at 37°C for 15 min. The reaction was stopped by addition of DMEM/F12 supplemented with 10% FBS, and the tissue was then mechanically dissociated with a Pasteur pipette. Cells were seeded into 96-well plates (1× 10^6^ cells/well) pre-coated with 0.01% poly-L-lysine, and cultured in Neurobasal Medium supplemented with 2% B27 and 100 U/ml penicillin, 100 μg/ml streptomycin at 37°C in 10% CO_2_.

The cells were seeded into plates and randomly assigned into six groups as described below containing six parallel samples per group. Control group (Group I), cells were first incubated with blank medium for 12 h, then incubated with the replaced blank medium for another 12 h. Model group (Group II), cells were first incubated with the medium containing Aβ_1–42_ (10 μM) for 12 h, then incubated with the replaced medium containing Aβ_1–42_ for another 12 h. Total lignans group (Group III-V), cells were first incubated with the medium containing Aβ_1–42_ (10 μM) for 12 h, then incubated with the replaced medium containing Aβ_1–42_ and TLS (100, 30, 10 μM crude extract of raw medicinal materials, respectively) for another 12 h. In Group VI (positive control), final concentration of donpesil in the assay was 20 μM. After treatments, the cells were assigned to determination of cell viability, analysis of mitochondrial membrane potential, expression of BACE1, Bal-2, JNK and p38.

### Determination of cell viability

Primary mouse neuronal cells were treated with TLS (100, 30, 10 μM), Aβ_1–42_ (10 μM) and donepezil (20 μM) for 48 h, and cell viability was detected by MTT assay. After drug treatment described above, the stock MTT solution was added to all wells to obtain a final concentration of 0.25 mg/ml, and plates were incubated for 4 h. The optical density (OD) values were measured at a wavelength of 492 nm. Morphological characteristics of primary cultured mouse neuronal cells were recorded by Nikon C2 plus Confocal (Nikon, Japan).

### Measurement of the mitochondrial membrane potential

Primary mouse neuronal cells were treated with TLS (100, 30, 10 μM), Aβ_1–42_ (10 μM) and donepezil (20 μM) for 48 h. The mitochondrial membrane potential (MMP) were assessed according to the alterations of fluorescence density upon Rhodamine 123 stain. The results were evaluated by morphological characteristics of primary cultured mouse neuronal cells.

### Assessment of BACE1, Bal-2, JNK and p38 expression

Primary neuronal cells were treated with TLS (100 μM), Aβ_1–42_ (10 μM) and donepezil (20 μM) for 48 h. The cells were washed with ice-cold PBS and centrifuged at 1000 rpm at 4°C for 10 min. The pellets were resuspended with 1000 μL of PBS, freeze-thawed twice at −20°C and centrifuged at 10,000 rpm at 4°C for 15min. The supernatant was collected for BACE-1, Bal-2, JNK and p38 assays, by means of the assay kits according to the manufacturer's directions.

### Statistical analysis

All statistical analyses were performed using the SPSS software, version 19.0. Results are expressed as mean ± S.E.M. (*n* = number of experiments). Data were obtained by applying the analysis of variance (ANOVA), followed by Tukey's multiple comparison test, *p* values < 0.05 were considered as significant.

## Results and Discussion

### Effects of TLS on the performance of Aβ_1-42_-induced mice in the locomotor activity test

Locomotor activity was designed to exclude the possibility that the changes on learning and memory were caused by the differences from animal itself. As shown in S1 Fig, there was no significant difference in the locomotor activity and the immobility among all the groups.

### Effects of TLS on the performance of Aβ_1-42_-induced mice in the shuttle-box test

**Fig 3:**
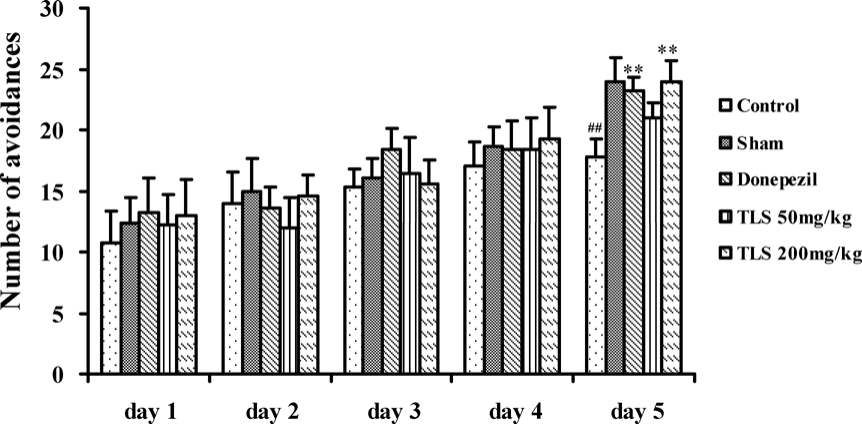
Effects of TLS on the performance of Aβ_1–42_-treated mice in the shuttle-box test. Sham and control groups: mice were given CMC-saline solution 0.2 ml/mouse, i.g.. Donepezil group: mice were given donepezil 3 mg/kg, i.g.. TLS 50 and 200 mg/kg group: mice were given 50, or 200 mg/kg of TLS, i.g., respectively. Values indicated mean ± S.E.M. and were analyzed by one-way analysis of variance (ANOVA) followed by Tukey's multiple comparison test (*n* = 12). ^**#**^*p* < 0.05, ^**##**^*p* < 0.01 compared with the sham group; *p < 0.05, **p < 0.01 compared with the control group.

On the acquisition session (from day 11 to day 14) of the shuttle-box test, the number of avoidance was increased following the test, and there was no significant difference among all the groups. On the post-training session (day 15), the number of avoidances in the control group was significantly less than that in the sham group (*p* < 0.01). The decreased avoidances induced by Aβ_1–42_ were significantly improved by donepezil (3 mg/kg, *p* < 0.01) and TLS (200 mg/kg, *p* < 0.01), however, there was no significantly different between the lower dose of TLS (50 mg/kg) treated group and control group (*p* = 0.126) (Fig 3).

### Effects of TLS on the performance of Aβ_1-42_-induced mice in the Morris water maze test

**Fig 4:**
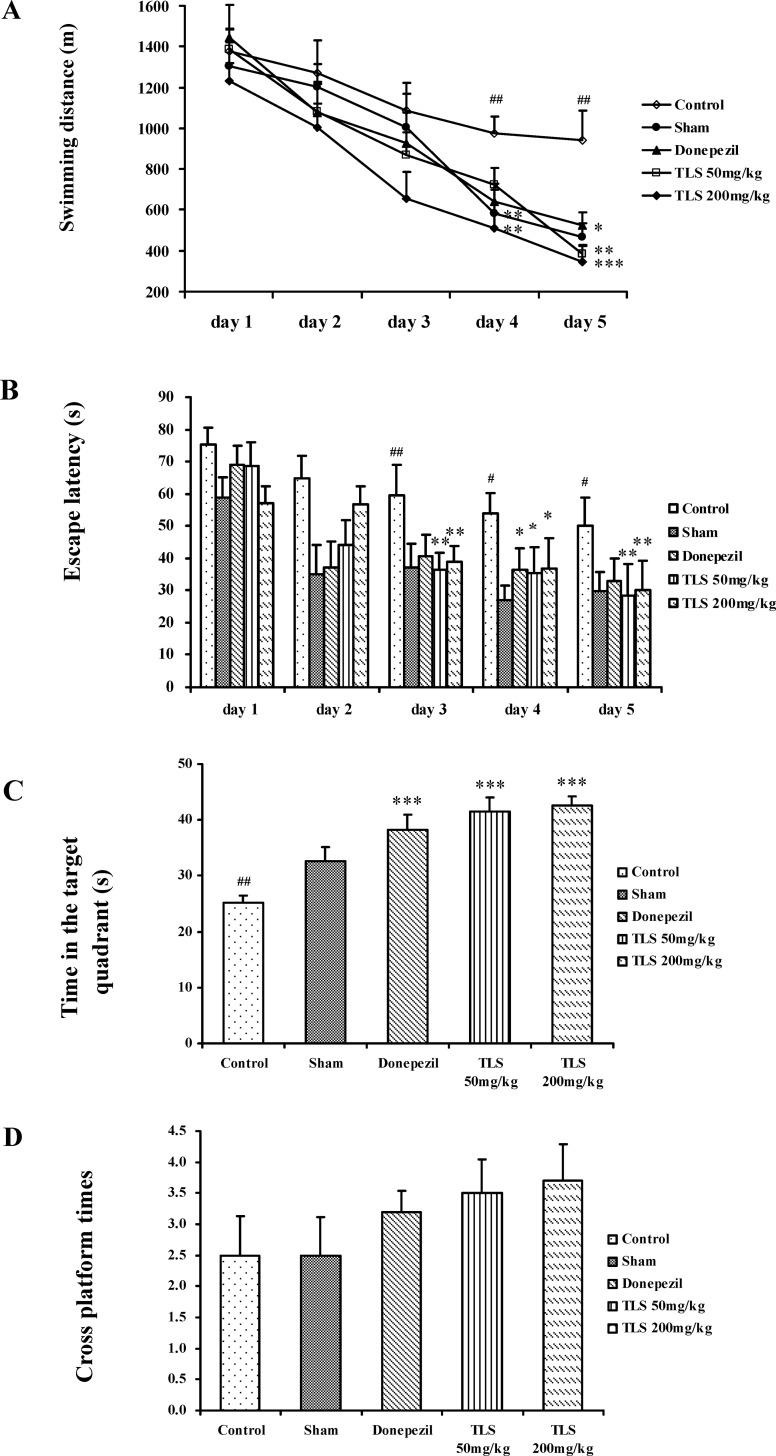
Effects of TLS on the performance of Aβ_1–42_-treated mice in the Morris water maze test. Swimming distance (A), Escape latency (B), Time in the target quadrant (C), and Cross platform times (D) were measured. Values indicated mean ± S.E.M. and were analyzed by one-way analysis of variance (ANOVA) followed by Tukey's multiple comparison test (*n* = 12). ^**#**^*p* < 0.05, ^**##**^*p* < 0.01 compared with the sham group; *p < 0.05, **p < 0.01 compared with the control group.

In the water maze test, the control group took more time and a longer swimming distance on the last three training days to reach the platform compared with the sham group in the place navigation test carried out from day 16 to day 20 after injection of Aβ_1–42_ (*p* < 0.05). As shown in Fig 4, the TLS treated groups significantly ameliorated the effect of Aβ_1–42_ on escape latency, and noticeably decreased the swimming distance. Donepezil also significantly ameliorated escape latency and swimming distance compared with the control group (Fig 4A and 4B). No significant difference was observed among all the groups in swimming speed throughout the test (S2 Fig).

Memory retention of the platform location was assessed in the spatial probe test performed on the day following place navigation test, mice in the control group spent less time in the target quadrant than the sham group (*p* < 0.01), but there was no difference in cross platform times (*p* = 1.00). The decreased swimming time in the target quadrant induced by Aβ_1–42_ was significantly restored by TLS 200 mg/kg (*p* < 0.001), TLS 50 mg/kg (*p* < 0.001) and donepezil 3 mg/kg (*p* < 0.001) (Fig 4C and 4D).

### Effects of TLS on the levels of MDA, T-AOC and activity of SOD in the hippocampus and cerebral cortex of mice

We evaluated the activity of SOD, T-AOC and the level of MDA in the hippocampus and cerebral cortex to elucidate whether Aβ_1–42_ injection makes any change in the antioxidant status within the brain or this change is reversible by the treatment of TLS. Compared with the sham group, the control group generated a dramatic decrease in the activity of SOD and T-AOC as well as a significant increase in MDA production in the hippocampus (*p* < 0.01, p < 0.05 and *p* < 0.01, respectively) and cerebral cortex (*p* < 0.01, respectively). However, both in the hippocampus and cerebral cortex, TLS 200 mg/kg treated group significantly restored the activity of SOD (*p* = 0.004, and *p* = 0.009, respectively) and T-AOC (*p* = 0.005, and *p* = 0.001, respectively). Donepezil and TLS 50 mg/kg treated groups also significantly improved the activities of SOD and T-AOC in the hippocampus and cerebral cortex. The increase in MDA production was markedly attenuated by the treatment with Donepezil or TLS (50, 200 mg/kg) (Table 2).

**Table 2:** Effects of TLS on the levels of MDA and the activities of SOD and T-AOC in the hippocampus and cerebral cortex.

Group	Hippocampus			Cerebral cortex		
	MDA (nmol/mg protein)	SOD (U/mg protein)	T-AOC (U/μg protein)	MDA (nmol/mg protein)	SOD (U/mg protein)	T-AOC (U/μg protein)
Control	16.63 ± 2.12 ^##^	19.26 ± 0.62 ^##^	6.06 ± 1.23 ^#^	18.13 ± 2.52 ^##^	24.03 ± 1.10 ^##^	51.36 ± 5.76 ^##^
Sham	11.47 ± 2.27	26.36 ± 1.17	7.95 ± 0.93	11.41 ± 1.15	34.18 ± 1.28	67.38 ± 3.73
Donepezil	13.52 ± 3.13	23.68 ± 1.29	7.80 ± 1.36 *	14.76 ± 2.01	35.90 ± 4.50 **	66.54 ± 3.21 *
TLS 50 mg/kg	14.06 ± 3.07	26.13 ± 1.83 *	7.84 ± 0.55 *	15.11 ± 0.79	30.02 ± 1.79	64.32 ± 4.11 *
TLS 200 mg/kg	12.08 ± 2.67 *	28.07 ± 2.65 **	8.41 ± 0.38 **	12.83 ± 1.87 *	32.84 ± 2.36 **	71.71 ± 1.72 **

Values indicated mean ± S.E.M. and were analyzed by one-way analysis of variance (ANOVA) followed by Tukey's multiple comparison test (*n* = 8).

^#^*p* < 0.05 and

^##^*p* < 0.01 compared with the sham group

*p < 0.05 and

**p < 0.01 compared with the control group.

### Effects of TLS on the levels of BACE1 and Aβ_1–42_ in the hippocampus of mice

**Fig 5:**
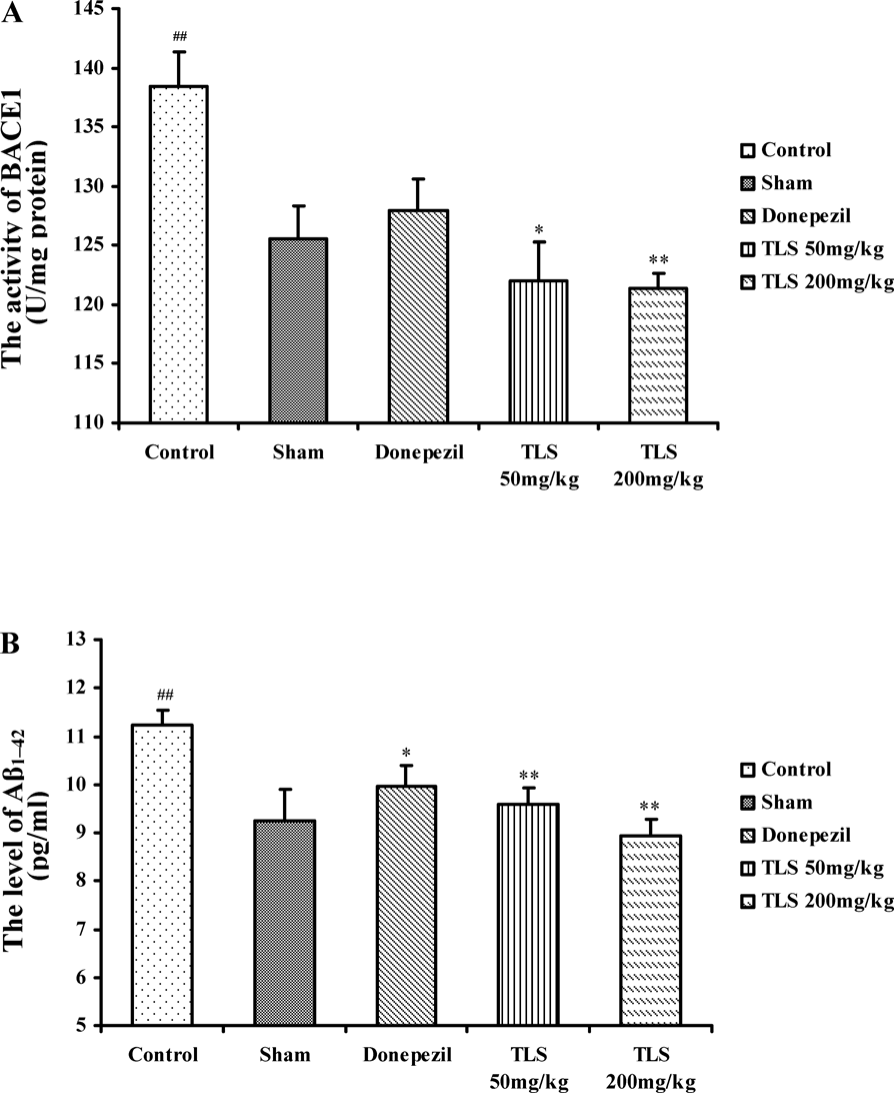
Effects of TLS on the expression of BACE1 and the level of Aβ_1–42_ in the hippocampus of Aβ_1–42_-treated mice. The expression of BACE1 (A), the level of Aβ_1–42_ (B). Values indicated mean ± S.E.M. and were analyzed by one-way analysis of variance (ANOVA) followed by Tukey's multiple comparison test (*n* = 8). ^**#**^*p* < 0.05, ^**##**^*p* < 0.01 compared with the sham group; *p < 0.05, **p < 0.01 compared with the control group.

As shown in Fig 5, the expression of BACE1 in the control group was significantly increased compared with the sham group (*p* = 0.019). However, TLS 50 mg/kg, and 200 mg/kg treated groups significantly restored the activity of BACE1 (*p* = 0.047, and *p* = 0.008, respectively). The level of Aβ_1–42_ in the control group was markedly higher than the sham group (*p* = 0.006). Nevertheless, the TLS 50 mg/kg, 200 mg/kg, and Donepezil treated groups generated a dramatic decrease on the level of Aβ_1–42_ (*p* = 0.003, *p* = 0.001, and *p* = 0.022, respectively).

We then tested the level of Aβ_1–42_ on the performance of behavioral tests. As a result, a significant (*p* = 0.018) negative correlation was observed between Aβ_1–42_ and the swimming time spent in the target quadrant in the water maze test. We have also investigated the relationship between Aβ_1–42_ and BACE1. No-significant (*p* = 0.322) correlation was observed between Aβ_1–42_ and BACE1.

### Effects of TLS on MAPKs inflammatory signaling pathways in the hippocampus and cortex of Aβ_1-42_-induced mice

**Fig 6:**
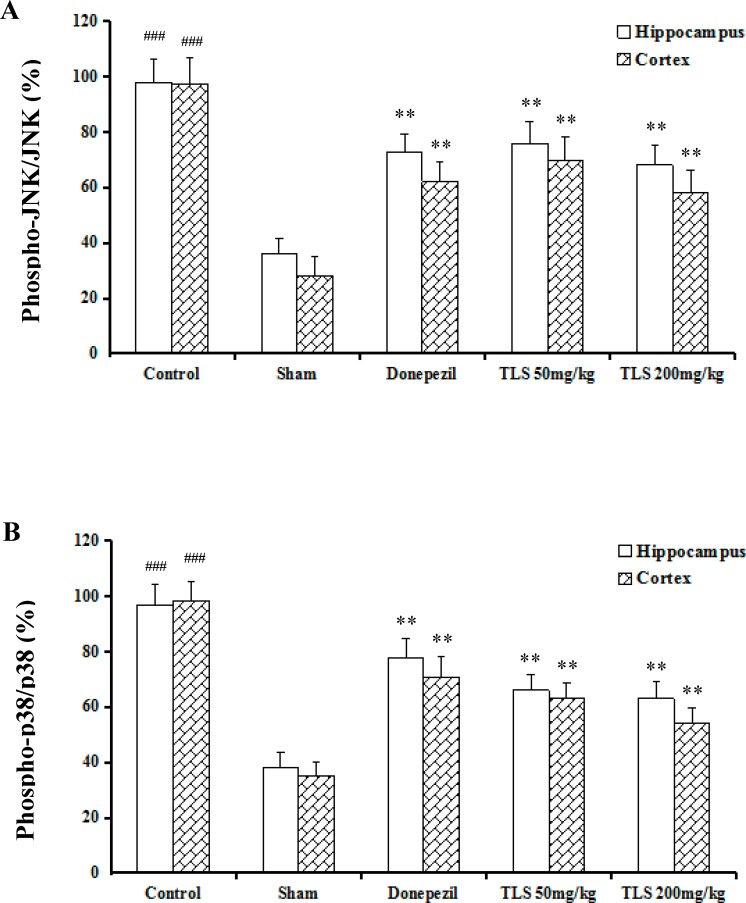
Effects of TLS on the MAPKs inflammatory signaling pathways in the hippocampus and cortex of Aβ_1–42_-treated mice. The expression of JNK (A), p38 (B). Values indicated mean ± S.E.M. and were analyzed by ANOVA followed by Tukey's multiple comparison test (*n* = 8). ^**#**^*p* < 0.05, ^**##**^*p* < 0.01, ^**###**^*p* < 0.001 compared with the sham group; *p < 0.05, **p < 0.01 compared with the control group.

The effects of TLS on the activation of MAPKs inflammatory signaling pathways were determined in Aβ_1-42_-mice. It was found that TLS (50, 200 mg/kg) significantly inhibited Aβ_1-42_-induced phosphorylation of JNK and p38, which both contributed to MAPKs signaling pathways, while their non-phosphorylated forms remained unchanged (Fig 6A and 6B), showing remarkable anti-inflammation effect.

### Effects of TLS on histopathological changes in the hippocampus of mice

**Fig 7:**
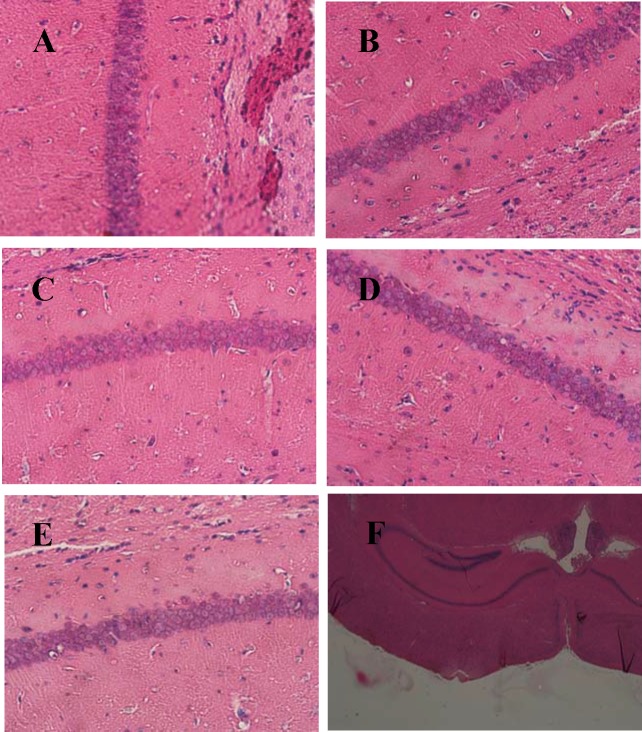
Effects of TLS on the histopathological changes in the hippocampus (CA1 region) of Aβ_1–42_-treated mice. Slice of hippocampus (A), Control group (B), Sham group (C), TLS 50 mg/kg group (D), Donepezil group (E), and TLS 200 mg/kg group (F). The magnification was 40×.

HE staining was performed to detect the neuronal integrity and orderliness in the hippocampus. The neuronal layers in the CA1 region of the hippocampus have shown rarefaction, disordered, pronounced shrinkage nuclei and swollen neuronal bodies in the control group (Fig 7B) compared with the sham group (Fig 7C). TLS 50 mg/kg treated group (Fig 7D), donepezil treated group (Fig 7E) and 200 mg/kg treated group (Fig 7F) significantly inhibited the histopathological damage, as those pictures shown the nucleoli were clearly visible, and there was no edema cell. The picture of entire hippocampus in Fig 7A did not show any inflammatory cells.

### Effects of TLS on cell viability of Aβ_1-42_-induced primary mouse neuronal cells

**Fig 8:**
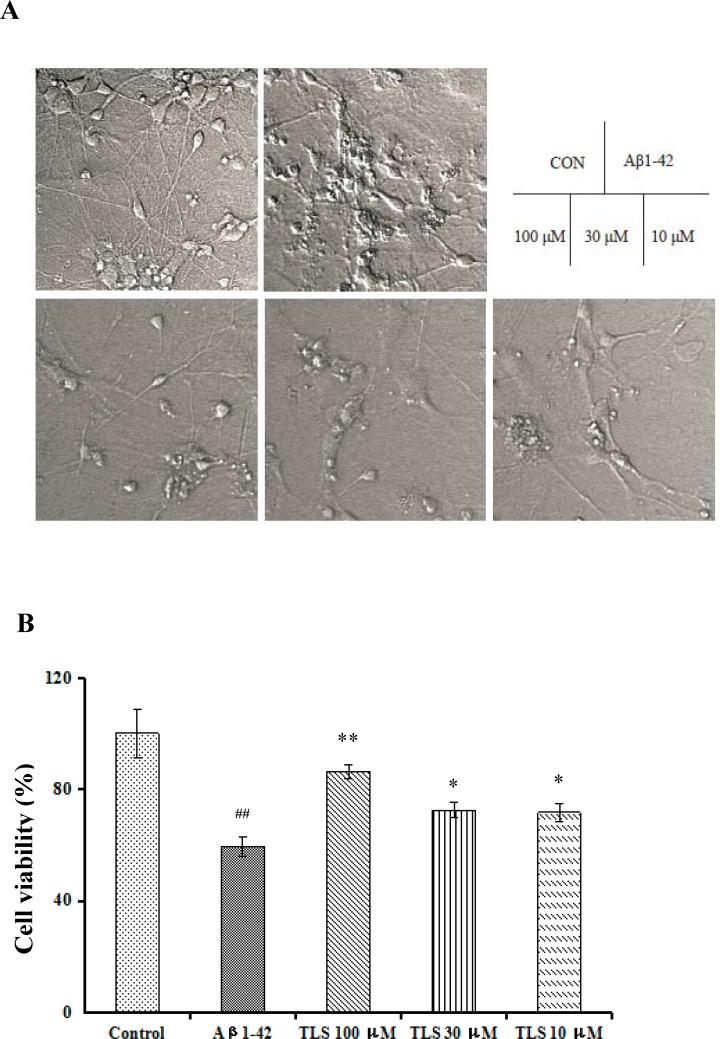
Effects of TLS on cell viability of the primary cultured mouse neuronal cells induced by Aβ1–42. Primary mouse neurons were treated with TLS (10–100 μM) and Aβ_1–42_ (10 μM) for 48 h. (A) Morphological characteristics of primary cultured mouse neuronal cells. (B) Cell viability was assessed by MTT assay. ^**#**^*p* < 0.05, ^**##**^*p* < 0.01 compared with the control group; *p < 0.05, **p < 0.01 compared with the Aβ_1–42_ group.

The cell viability of primary mouse cultured neuronal cells induced by Aβ_1–42_ was measured with MTT to elucidate the ameliorate effects of TLS. Compared with the control group, the Aβ_1–42_ group generated a dramatic decrease in the OD value (*p* < 0.01). However, TLS (100, 30, 10 μM) treated group significantly restored the decrease in cell viability (*p* < 0.01, *p* < 0.05, *p* < 0.05, respectively). The cell viability reached up to 86.3 ± 2.5%, 72.51 ± 2.65% and 71.60 ± 3.04%, when cells were treated with 100, 30 and 10 μM TLS, respectively (Fig 8B). Morphological characteristics of primary cultured mouse neuronal cells were shown in Fig 8A. In control group, distribution of the cells was uniform, with clear cell body, and the synapse of the cell was connected into a dense reticulation. The cells in model group aggregated, pronounced shrinkage cell body, with fuzzy edge blur. Synapse fractured, reduced or even disappeared. However, treatment of TLS improved the aggregation of cells and axonal fracture induced by Aβ_1–42_.

### Effects of TLS on the alterations of MMP in Aβ_1-42_-induced primary mouse neuronal cells

**Fig 9:**
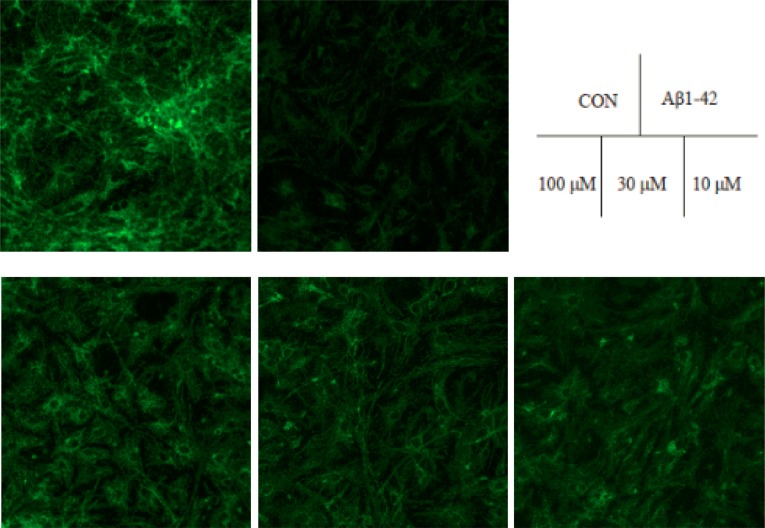
Effects of TLS on the alterations of MMP in Aβ_1-42_-induced primary mouse neuronal cells. Primary mouse neurons cells were treated with TLS (10–100 μM) and Aβ_1–42_ (10 μM) for 48 h. Morphological characteristics of primary cultured mouse neuronal cells.

Effect of TLS on the changes of MMP in primary cultured mouse neuronal cells were determined according to the alterations of fluorescence density upon Rhodamine 123. Compared with the control group, Aβ_1–42_ treatment generated a decrease of fluorescence intensity, leading to an oxidation effect on the cells. However, TLS (100, 30, 10 μM) treated group significantly increased the MMP, indicated that TLS could protect the primary neuronal cells by blocking the decrease of MMP (Fig 9).

### Effects of TLS on the expression of BACE1 in Aβ_1-42_-induced primary mouse neuronal cells

**Fig 10:**
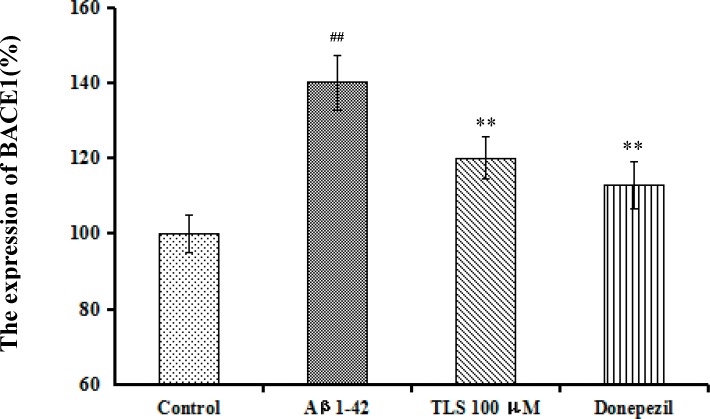
Effects of TLS on the expression of BACE1 in Aβ_1-42_-induced primary mouse neuronal cells. Values indicated mean ± S.E.M. and were analyzed by one-way analysis of variance (ANOVA) followed by Tukey's multiple comparison test (*n* = 6). ^**#**^*p* < 0.05, ^**##**^*p* < 0.01 compared with the control group; *p < 0.05, **p < 0.01 compared with the Aβ_1–42_ group.

The expression of BACE1 in the model group was significantly increased compared with the control group (*p* < 0.01, Fig 10). However, treatment of TLS 100 μM significantly restored the activity of BACE1 in Aβ_1-42_-induced primary mouse neuronal cells (*p* < 0.01), showing similar effect as in the model mice.

### Effects of TLS on the level of Bcl-2 in Aβ_1-42_-induced primary mouse neuronal cells

**Fig 11:**
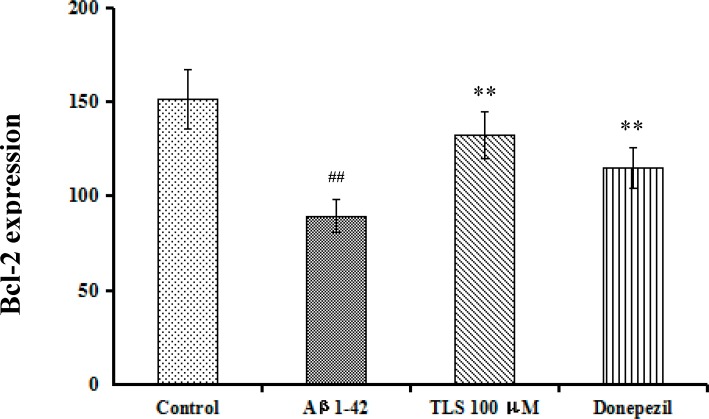
Effects of TLS on the level of Bcl-2 in Aβ_1-42_-induced primary mouse neuronal cells. Values indicated mean ± S.E.M. and were analyzed by ANOVA followed by Tukey's multiple comparison test (*n* = 6). ^#^*p* < 0.05, ^##^*p* < 0.01 compared with the control group; *p < 0.05, **p < 0.01 compared with the Aβ_1–42_ group.

The anti-apoptotic effects of TLS on the level of Bcl-2 were also examined. A significant decrease of protein expression level of Bcl-2 was found in the Aβ_1-42_-induced group compared with the control group (*p* < 0.01), however, TLS (100 μM) treated cells had significantly higher Bcl-2 level than those in model group (Fig 11). These results demonstrated that TLS treatment could reduce proapoptotic molecules in the primary mouse neuronal cells induced by Aβ_1–42_, and showed significant anti-apoptotic effects.

### Inhibition effects of TLS on MAPKs inflammatory signaling pathways in Aβ_1-42_-induced primary mouse neuronal cells

**Fig 12:**
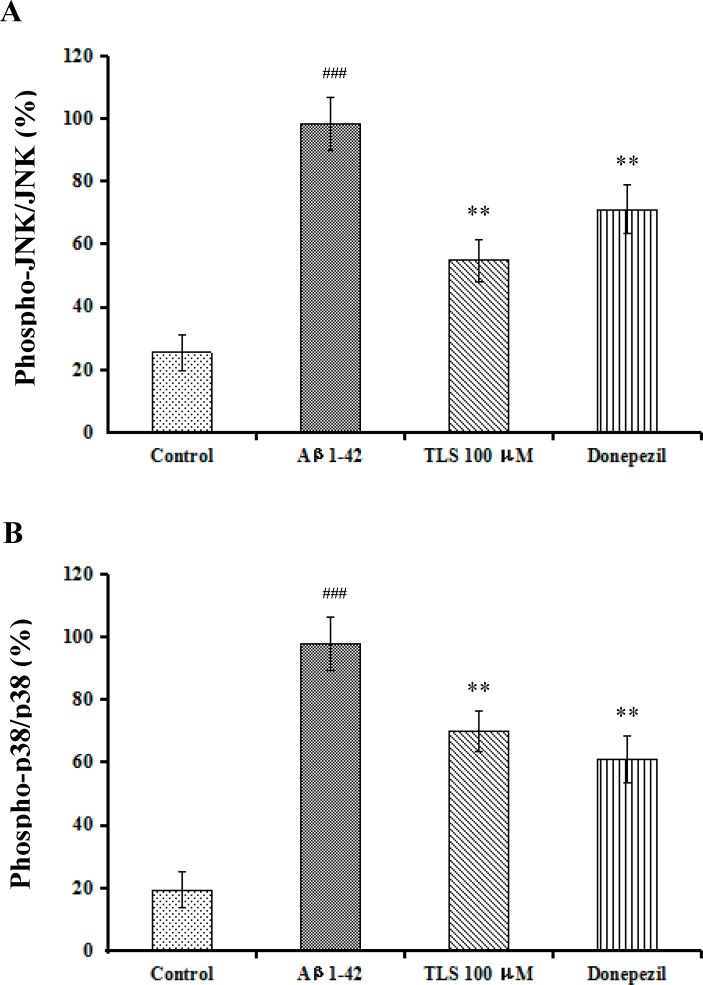
Effects of TLS on the expressions of JNK and p38 in Aβ_1-42_-induced primary mouse neuronal cells. The expression of JNK (A), p38 (B). Values indicated mean ± S.E.M. and were analyzed by ANOVA followed by Tukey's multiple comparison test (*n* = 6). ^#^*p* < 0.05, ^##^*p* < 0.01 compared with the control group, ^**###**^*p* < 0.001 compared with the sham group; *p < 0.05, **p < 0.01 compared with the Aβ_1–42_ group.

The effects that TLS exerted on the activation of MAPKs inflammatory signaling pathways were determined in Aβ_1-42_-induced primary mouse neuronal cells. Consistent with the results in the mice, TLS (100 μM) significantly inhibited Aβ_1-42_-induced phosphorylation of JNK and p38 (Fig 12A and 12B), which indicated anti-inflammation effect of TLS both *in vitro* and *in vivo*.

### Discussion

The *in vivo* study indicated that TLS significantly alleviated cognitive deficits validated by the behavioral tests in Aβ_1–42_-induced mouse models of Alzheimer’s disease. The improvement of the activities of SOD, T-AOC, and the decrease on the levels of MDA, BACE1 and Aβ_1–42_ indicated that TLS could restore the biochemical pathological changes. The results of histopathological in the hippocampus proved that TLS noticeably ameliorated the pathological changes induced by Aβ_1–42_ in mice. The results of animal-relevant study suggested that TLS might protect against cognitive deficits and neurodegeneration by releasing the damage of oxidative stress, decreasing the level of Aβ_1–42_ and inhibiting the expression of BACE1.

AD is probably caused by complex interactions, the pathogenic mechanisms underlying AD include impaired cholinergic function, increased oxidative stress, overproduction of Aβ peptides, expression of inflammatory mediators and so on [27]. However, compelling evidences suggested that the overproduction of Aβ peptides, especially Aβ_1–42_, oxidative stress and apoptosis induced by Aβ play a critical role in the progressive of AD [28]. Mice intracerebroventricular injected with Aβ can mimic some cognitive deficits in AD, and this model is very economic and reliable. In the current study, we tested the infusion of Aβ_1–42_ into the lateral ventricle, and investigated some important biochemical parameters about oxidative stress and cell damage, and the results were conformed to the previous studies [29–32].

MDA is one of the main outcomes of lipid peroxidation, which directly damages cell membranes [33]. T-AOC is an indicator of total intracellular antioxidant status. SOD is an important enzyme in the oxidation system, which protected cellular against damage caused by oxygen-derived free radicals. T-AOC and SOD not only represent the anti-oxidant property, but also reflect cell damage indirectly, and can be used to determine the degree of cell damage [34, 35]. In our experimental system, mice in the control group became amnesia, and the biochemical parameters of oxidative stress and cell damage were changed. The results demonstrated that a common oxidative stress mechanism is responsible for the toxicity of Aβ, which corroborating previous studies [36–38]. Moreover, the results of behavioral tests have been related to the level of Aβ_1–42_ and the activity of β-secretase (BACE1). The significant negative correlation between the swimming time in the target quadrant and the level of Aβ_1–42_ was prettily demonstrated that the level of Aβ_1–42_ in the hippocampus directly affected learning and memory. BACE1 cleavage has been proved mainly occurs in the endosome [39], in this study, the activity of BACE1 was significantly increased in the control group, while no-significant correlation was found between the level of Aβ_1–42_ and the activity of BACE1 in the hippocampus. Therefore, we presumed that γ-secretase cleavage of APP, the other path of Aβ outcome, was also existed in the endosome, which should be explored in further research.

To reveal the potential mechanisms of TLS-induced effects not only in model animals but also in cells, we further investigate the anti-apoptotic and anti-inflammatory effects of TLS on the Aβ_1–42_-induced primary mouse neuronal cells. Overall, pretreatment with TLS markedly inhibited cell death induced by Aβ_1–42_ exposure as determined by cell viability assay with no toxicity to the cells. In primary neuronal cells, TLS (10–100 μM) significantly blocked the decrease of MMP induced by Aβ_1–42_, showing anti-oxidation effects on primary neuronal cells.

The anti-apoptotic effects of TLS on the level of Bcl-2 were also measured. It is well-known that apoptosis is a kind of active cell death controlled by a discrete set of genes such as Bcl-2, Bad and Bax. Each alteration on expressions of these genes may change the result of apoptosis. Bcl-2 family proteins, are important regulators in the mitochondria apoptosis pathway and take part in lymphocytes apoptosis and homeostasis [40, 41]. According to our results, TLS treated cells showed significantly higher Bcl-2 levels than those in model group, demonstrated that TLS treatment could reduce proapoptotic molecules in the primary mouse neuronal cells induced by Aβ_1–42_, and showed significant anti-apoptotic effects.

Furthermore, the effects of TLS exerted on the activation of MAPK family was determined. The MAPK family is involved in the induction of proinflammatory cytokines and chemokines, tightly associated with oxidative stress and inflammation [42]. It was found that TLS significantly decreased the expression of JNK and p38 signaling pathways in mice and primary mouse neuronal cells, by inhibiting the phosphorylation of JNK and p38, indicating remarkable anti-inflammatory effects both *in vivo* and *in vitro*.

## Conclusion

The present study demonstrated that total lignans of *Schisandra chinensis* potentially reversed alterations in cognitive behavioral, biochemical and histopathological changes induced by Aβ_1–42_ *in vivo* or *in vitro*. These beneficial effects of TLS might be attributed partly by releasing the damage of oxidative stress, inhibiting the expression of BACE1 and the MAPKs inflammatory signaling pathways. Our findings suggested that TLS might be a worthwhile adjunct in the treatment of cognitive and behavioral deficits.

## Supporting Information

S1 Fig Effect of TLS on the performance of Aβ_1–42_-treated mice in the locomotor activity test. Sham and control groups: mice were given CMC-saline solution 0.2 ml/mouse, i.g.. Donepezil group: mice were given donepezil 3 mg/kg, i.g.. TLS 50 mg/kg, and TLS 200 mg/kg group: mice were given 50, or 200 mg/kg of TLS, i.g., respectively. Locomotor activity (A), Immobility (B) were measured. Values indicated mean ± S.E.M. and were analyzed by one-way analysis of variance (ANOVA) followed by Tukey's multiple comparison test (*n* = 12). *p < 0.05 compared with the control group; ^#^*p* < 0.05 compared with the sham group.
(TIF)

S2 Fig Swimming speed of Aβ_1–42_-treated mice in the Morris water maze test. Sham and control groups: mice were given CMC-saline solution 0.2 ml/mouse, i.g.. Donepezil group: mice were given donepezil 3 mg/kg, i.g.. TLS 50 mg/kg, and TLS 200 mg/kg group: mice were given 50, or 200 mg/kg of TLS, i.g., respectively. Values indicated mean ± S.E.M. and were analyzed by one-way analysis of variance (ANOVA) followed by Tukey's multiple comparison test (*n* = 12).
(TIF)
