# Supplementary figures and images for: Total Lignans of Schisandra chinensis Ameliorates Aβ1-42-Induced Neurodegeneration with Cognitive Impairment in Mice and Primary Mouse Neuronal Cells
Source: PLoS One. 2016 Apr 1;11(4):e0152772. doi: 10.1371/journal.pone.0152772 (PMC4818042; doi:10.1371/journal.pone.0152772)

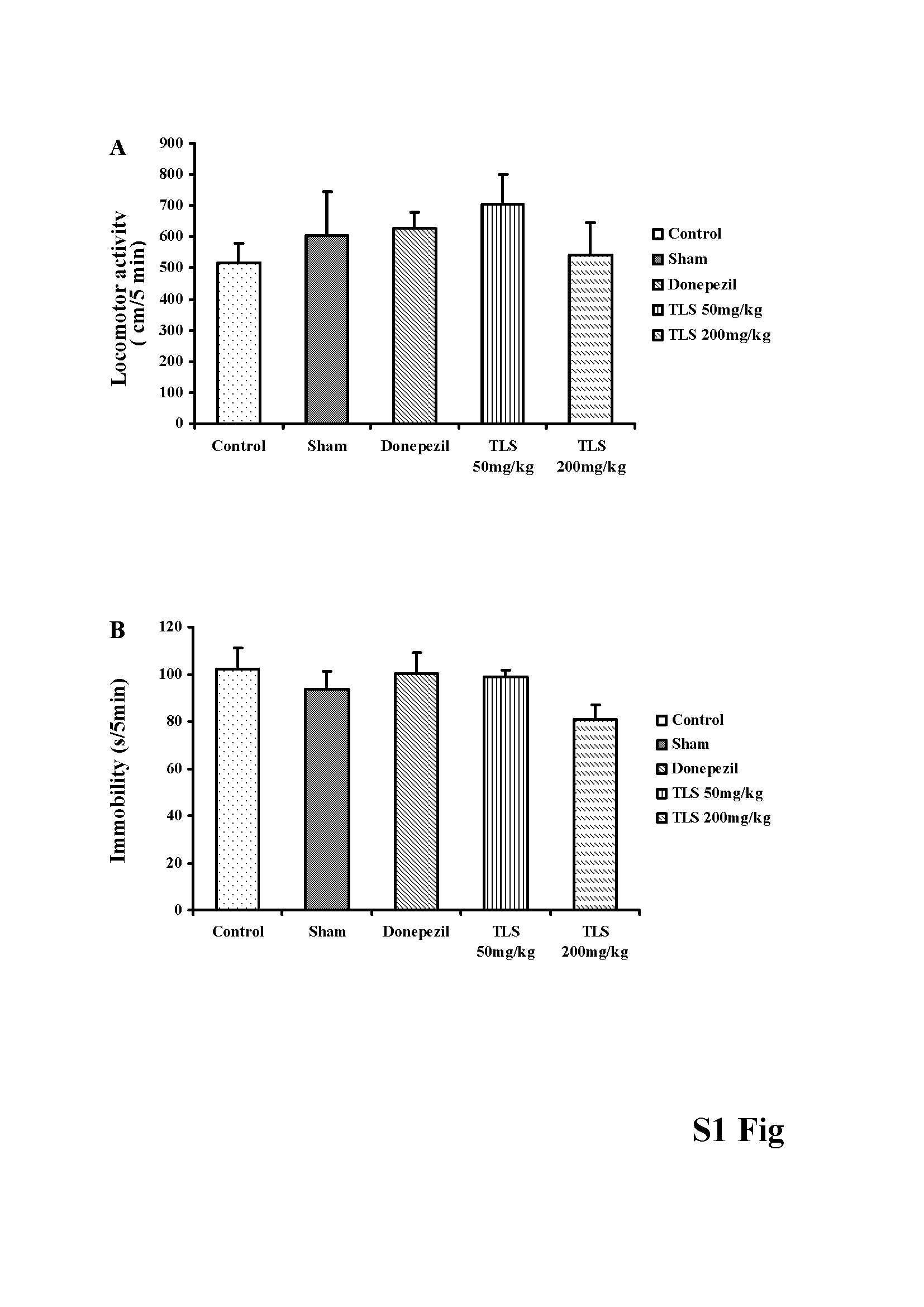

Supplement: S1 Fig — Sham and control groups: mice were given CMC-saline solution 0.2 ml/mouse, i.g.. Donepezil group: mice were given donepezil 3 mg/kg, i.g.. TLS 50 mg/kg, and TLS 200 mg/kg group: mice were given 50, or 200 mg/kg of TLS, i.g., respectively. Locomotor activity (A), Immobility (B) were measured. Values indicated mean ± S.E.M. and were analyzed by one-way analysis of variance (ANOVA) followed by Tukey's multiple comparison test (n = 12). *p < 0.05 compared with the control group; #p < 0.05 compared with the sham group. (TIF) [file pone.0152772.s001.tif]

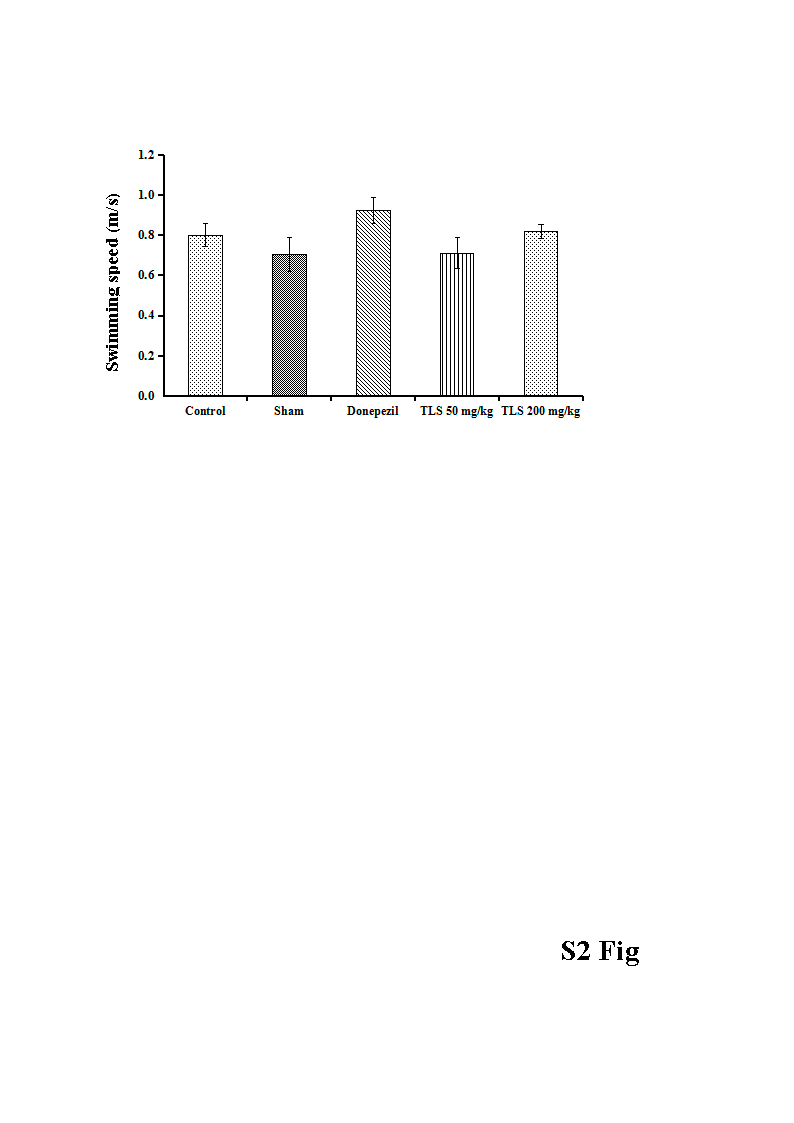

Supplement: S2 Fig — Sham and control groups: mice were given CMC-saline solution 0.2 ml/mouse, i.g.. Donepezil group: mice were given donepezil 3 mg/kg, i.g.. TLS 50 mg/kg, and TLS 200 mg/kg group: mice were given 50, or 200 mg/kg of TLS, i.g., respectively. Values indicated mean ± S.E.M. and were analyzed by one-way analysis of variance (ANOVA) followed by Tukey's multiple comparison test (n = 12). (TIF) [file pone.0152772.s002.tif]
